# Knowledge, attitude and practice of family caregivers on pressure injury prevention for community-dwelling older adults: a cross-sectional study in an Indonesia City

**DOI:** 10.1186/s12912-024-02662-w

**Published:** 2025-01-07

**Authors:** Sheizi Prista Sari, Irma H. J. Everink, Christa Lohrmann, Yufitriana Amir, Eka Afrima Sari, Ruud J. G. Halfens, Jos M. G. A. Schols

**Affiliations:** 1 Faculty of Nursing, Universitas Padjadjaran, Jl. IR. Soekarno KM. 21 Jatinangor, West Java, Bandung, Indonesia; 2 Department of Health Services Research, Care and Public Health Research Institute (CAPHRI), Maastricht University, Maastricht, The Netherlands; 3 Department of Nursing Science, Medical University of Graz, Graz, Austria; 4 Faculty of Nursing, Universitas Riau, Pekanbaru, Indonesia; 5 Department of Family Medicine, Care and Public Health Research Institute (CAPHRI), Maastricht University, Maastricht, The Netherlands

**Keywords:** Knowledge, Attitude, Practice, Family caregivers, Pressure Injury, Prevention, Community-Dwelling older adults

## Abstract

**Background:**

Older adults in Indonesia are mostly living at home with their families. Informal care provided by family caregivers is essential to prevent older adults from getting pressure injuries (PIs). The objectives of this study were to examine the knowledge, attitude and practice of family caregivers regarding PI prevention among community-dwelling older adults in Indonesia.

**Methods:**

A cross-sectional survey was conducted involving 267 family caregivers randomly selected from a population list provided by municipalities in Bandung, West Java, Indonesia. The study utilized the paper-based Knowledge, Attitude, and Practice on Pressure Injury (KAP-PI) questionnaire. Descriptive analyses (i.e. percentage, mean and standard deviation) were used to present participants’ scores in each domain based on their characteristics or categories and scores in every single item of questions or statements. Bivariate comparison analyses were conducted using independent t-test or ANOVA test to compare scores and a Chi-square was run to check correlations between knowledge, attitude and practice domains.

**Results:**

This study show that more than half of the participants (61.0%) had insufficient knowledge about PI prevention, but nearly all participants (94.4%) had a positive attitude toward PI prevention for their older relatives. Still, 41.2% of the participants had inadequate practice on PI prevention. A correlation analysis revealed that having sufficient knowledge and a positive attitude towards PI prevention were positively correlated with practice (*p* < .05).

**Conclusion:**

This study highlights a significant gap in family caregivers’ knowledge and practice regarding pressure injury (PI) prevention, despite their generally positive attitudes toward the care of older relatives. The findings are the initial diagnosis to guide educational strategies. Research should be performed to explore effective educational materials and an education program and also the necessary professional support to strengthen family caregivers’ role in PI prevention.

## Introduction

A pressure injury (PI) is defined as a localized damage to the skin and or underlying tissue, which usually occurs over a bony prominence due to pressure or pressure combined with shear [1]. In the last few decades, various international studies on the prevalence and incidence rates of pressure injuries (PIs) among this vulnerable group were published. The prevalence of PIs among older adults living in the community is estimated to range from 8 to 11%, with variations depending on geographic location, healthcare access, and the specific population studied [2–8], indicating that PIs remain a serious issue among community-dwelling older adults in countries around the world. The incidence continues to rise due to aging populations and increasing mobility limitations [9].

PI are often preventable if the right preventive measures are taken. Performing preventive measures is especially important among older adults who have a (high) risk of getting PIs, such as those with limited mobility due to various diseases (e.g. stroke) [7, 10, 11] or older adults with decreased activity-related activities of daily living, e.g. bathing, toileting and transferring [7, 10, 12], as well as also older adults with nutritional problems as malnutrition can cause problems related to repair and maintenance of tissue viability [13]. Studies have shown that involving families in PI prevention could decrease the PI prevalence and may reduce health care utilization [14–16].

In Indonesia, PIs among community-dwelling older adults also have become a critical public health issue. A study conducted by Sari et al. in Bandung, Indonesia, reported a prevalence rate of 11% for PIs among older adults living at home [10]. Considering that the majority of older adults in Indonesia live at home with their families [17], this finding underscores the urgent need for targeted prevention and care strategies that focus on the informal care provided by family caregivers. In addition, even though health care services are basically accessible, those who are at risk of PIs do not always access formal care [10, 18] due to various reasons, such as they prefer to self-medicate (60%), or think that they do not need formal health services (33%) [18]. As a consequence, family caregivers in Indonesia spend more time in PI prevention for community-dwelling older adults than formal health care professionals do, irrespective of what they do is correct or not [3, 10, 19, 20]. Therefore, family caregivers play a pivotal role in preventing and managing pressure injuries among Indonesian community-dwelling older adults.

To improve family caregivers’ competences on PI prevention, it is important to know what family caregivers know about PIs and about PI prevention (knowledge), and to know more about their attitude towards PI prevention. Also, it is important to get insight into the extent to which they already provide specific care to prevent PIs among their older relatives. The theoretical framework for this study rests on the understanding that effective pressure injury prevention is primarily dependent on the knowledge, attitude, and practices of caregivers [21, 22]. Having sufficient knowledge and skills for providing adequate PI prevention is essential [11, 23]. Also, research has shown that not only knowledge but also having a positive attitude towards PI prevention is utmost importance, as having a right attitude is a precursor for good caring behavior [24, 25]. Both knowledge and attitudes build a strong practice [21, 26, 27].

However, family caregivers’ knowledge, attitudes, and practices regarding pressure injury prevention are not well studied and their awareness and practices regarding pressure injury prevention remain under-researched. In addition, family members tend to receive little information or training on PI prevention [10, 12, 28, 29]. To the best of our knowledge, no studies are available that assess the knowledge and attitude of family caregivers of Indonesian community-dwelling older adults regarding PI prevention. Furthermore, no studies are available that evaluate if PIs among community-dwelling older adults in Indonesia are currently being prevented by family caregivers in the right way. This information is required to develop and deliver an effective and supportive educational program and also the need for professional support [22]. Therefore, this study aims to fill this knowledge gap by examining the Indonesian family caregivers’ knowledge, attitude and practice on PI prevention. The relationships between knowledge of, attitude toward, and preventive practice are also discussed.

## Methods

### Design

In this study, a cross-sectional survey was used among family members of community-dwelling older adults in Bandung, the capital city of West Java Province in Indonesia.

### Population and setting

The population of this study involved family caregivers of community-dwelling older adults in Bandung, Indonesia. These family caregivers (spouses, children, nieces, nephews, sisters or brothers) were informal caregivers providing care for their relatives aged 60 years or older – which are considered older adults in Indonesia [30]. The authors, SPS and EAS, collaboratively ensured a randomized selection process by choosing participants from a list of families provided by the municipalities in each region of Bandung. The list was randomly generated using numeric random software, which minimized the potential for bias. This approach ensured that every family had an equal chance of being selected, enhancing the representativeness and reliability of the sample. The number of family caregivers to be included in each region was determined by the ratio of older adult families to the total number of older adult families in all regions. Slovin’s Formula with the estimation error of 6% was used to calculate the ideal sample size [31]. The population of older adults (aged ≥ 60 years) in Bandung was 208,838 people [32], of which approximately 187,955 people (90%) live at home with their families [33]. Based on the estimation number of families with older adult(s), the sample size required was 267 family caregivers.

#### Inclusion criteria

The inclusion criteria for this study were family caregivers aged 18–59 years and caring for their older relative at home, irrespective of whether they live in the same house or not. They were responsible for the older adult(s) and were the ones who spent the most time caring for them. Family caregivers aged 60 years or above were excluded from the study because they were calculated as community-dwelling older adults instead of family caregivers.

### Measurement instrument

To ensure the validity of the tool used in this study, authors conducted a thorough search of available instruments that could address the study’s objectives. After reviewing several tools, we selected the paper-based self-administered Knowledge, Attitude, and Practice on Pressure Injury (KAP-PI) questionnaire, which has been tested in related research [34]. The KAP-PI instrument was created following best practices for developing and validating scales for health, social, and behavioral research [35], intentionally developed and validated in a population of Indonesian family caregivers of community-dwelling older adults, ensuring that it is well-suited for the objectives of this study. The detailed components and the psychometric properties of the KAP-PI questionnaire is provided below, as published in the original study [34]:

#### Characteristics of family caregivers

The characteristics of family caregivers addressed in the KAP-PI comprise demographic characteristics and caring characteristics. Demographic characteristics include age, gender, educational background (primary, lower secondary, upper secondary, short-cycle tertiary, and bachelor or equivalent), occupation (student, unemployed, employed, and self-employed), living arrangement (living in the same house with the older adult, or living in a different house), and relationship with the older adult (children vs. other relatives; brother, sister, nephew or niece). The caring characteristics include questions on (1) responsibility in caring for the relative (single or shared responsibility); (2) duration of caring for the relative (number of years), (3) number of hours per day support provided by the participant with ADL of their relative; (4) the presence or absence of PIs in the relative; and (5) whether or not the participant had experience in PI care.

#### Knowledge domain

The knowledge domain in the KAP-PI questionnaire consists of three topics, including 12 items in total: (1) ‘Definition of PI and older adults’ characteristics’ (2 items); (2) ‘Symptoms, cause(s), and consequences of PIs (3 items); and (3) ‘Prevention of PIs’ (7 items). All items have three multiple-choice answers with only one correct answer. An example item is " A thing that should be done when an older persons’ skin turns red is?” with the multiple-choice answer options: a) Let it dry itself; b) Release pressure and shear; c) Apply betadine or iodine. The answers on the knowledge domain were recorded as correct (‘1’) or wrong (‘0’) to obtain a total score which was presented as a percentage of the maximum possible score. For the scoring of this domain, the cut of point 65% was used to categorize participants into insufficient and sufficient knowledge groups. The KAP-PI questionnaire in the knowledge domain had excellent psychometric properties (an average of item difficulty of 0.57, a discriminating index of 0.59, quality of response alternatives of 0.20 and a Cronbach’s alpha of 0.83) [34].

#### Attitude domain

The attitude domain of the KAP-PI questionnaire consists of nine statements regarding PI prevention in community-dwelling older adults, such as “*I have to pay attention to the skin moisture and hygiene of the older relative in my house*”. Participants were asked to indicate to what extent they agree with the statements on a four-point Likert scale, ranging from ‘strongly disagree’ (1) to ‘strongly agree’ (4). In the attitude domain, the sum score can range between 9 and 36. A higher sum score indicates a more positive attitude towards PI prevention. For scoring, participants with a total score of 27 and above were considered to have a positive attitude towards PI prevention. Cronbach’s alpha of the KAP-PI instrument in the attitude domain was 0.93 [34].

#### Practice domain

The practice domain of the KAP-PI questionnaire consists of 12 statements on basic support (4 items), reposition and mobilization support (4 items), skin hygiene and moisture support (4 items) and practice on nutrition and hydration care (2 items). An example of one of the statements is: “*I Moisturize the skin of the older relative in my house by giving lotions/oils* “. All statements in the practice domain have a response option on a Likert scale, ranging from: (1) ‘never’ if the activity has never been done; (2) ‘sometimes’ if the activity is done 1 to 3 days per week; (3) ‘often’ if the activity is done 4 to 6 days per week; and (4) ‘always’ if the activity is done every day. In the practice domain, the sum score can range between 12 to 48. A higher sum score indicates a better PI practice. Based on this tool, participants with a total score of 36 and above in the practice domain were categorized as adequate practice on PI prevention. The internal consistency of the KAP-PI instrument in the practice domain had a Cronbach’s alpha of 0.89, indicating high reliability [34].

### Data collection

Data collection took place in the participants’ homes. Six trained research assistants who were nurses visited the selected participants. They explained the study’s objectives, the procedure, the anonymized use of data, and the right to stop participation at any moment. The selected participants were offered a free health consultation about their condition and the condition of older adult(s) whom they took care after filling the questionnaire. Participants who agreed to participate signed the informed consent form and completed the KAP-PI questionnaire in front of the research assistants. The completed instruments were collected directly by the enumerators and given to author SPS.

### Data analysis

Data were analyzed using International Business Machines Statistical Package for the Social Sciences (IBM SPSS) Statistics 26 (IBM Corp, Armonk, NY). Descriptive analyses (i.e. percentage, mean and standard deviation) were used to present participants’ scores in each domain based on their characteristics or categories and scores in every single item of questions or statements. Based on the tool used, the total score for each participant in each domain was divided into categories. Bivariate comparison analyses were conducted using either an independent t-test or ANOVA test to compare scores. Finally, Chi-square tests were used to examine the associations between knowledge, attitude, and practice scores, and the results were interpreted based on the significance levels obtained. An alpha of 0.05 was used.

### Ethics approval and consent to participate

All methods were performed in accordance with the relevant guidelines and regulations of the Declaration of Helsinki, a statement of ethical principles which directs research involving human subjects. This study received ethical approval from The Research Ethics Committee Faculty of Medicine, Universitas Padjadjaran Bandung (No. 138 / UN6.KEP/EC/2020). Two governmental institutions that have responsibilities in health care and community protection approved the research project before undertaken (the Indonesian Health Care Agency #070/13472-Dinkes and the National Unity Agency, Politics and Protection of the Regional People #070/3177/Bakesbangpol). Participants received information about the study and signed consent if they agreed to participate. Informed consent was obtained for experimentation with human subjects. Participants were not obligated to participate and could refuse participation before and during the data collection.

## Results

### Participants characteristics

In total, 267 family caregivers participated in this study. The response rate was 100%, meaning that all randomly selected family caregivers who met the inclusion criteria gave informed consent and completed the questionnaire. This high response rate was achieved because all participants were visited personally by two research nurses. The mean age of the participants was 37.2 years. The majority of participants was female (90.3%; *n* = 241), married (86.1%; *n* = 230) and had a low educational background (86.1% lower secondary education). More than half of the participants (60.3%; *n* = 161) had a job. Only 22.8% (*n* = 61) of the participants were children of the older adults, the rest involved other relatives of older adults (i.e. niece, nephew, sister or brother). Most participants lived with the older adult in the same house as an extended family member (93.6%; *n* = 250), and shared responsibility with other family members in caring for the older adult (86.4%; *n* = 226). In total, 23% (*n* = 61) of the participants cared for older adults for more than five years. However, almost half of the participants (43.4%; *n* = 116) could not define how many hours they supported the older adult’s activity daily living in a day. About 11% (*n* = 30) of the participants lived with older adults who suffered from (a) PI(s), and only 13% (*n* = 4) of them had experience in taking care of people suffering PIs. The demographic data of all participants and their caring activities are shown in Table 1.

**Table 1:** Participants’ characteristic of and results on the total score of each domain (*n* = 267)

Participant’s characteristics	Participants		Knowledge		Attitude		Practice	
	*n*	%	Mean % (SD)	Difference	Mean (SD) (Range = 9–36)	Difference	Mean (SD) (Range = 12–48)	Difference
Total	267	100%	52.8 (24.8)		30.2 (3.9)		37.9 (8.0)	
Gender								
Male	26	9.7	52.2 (24.8)	t = − .12^a^ *p* = .90	30.2 (3.5)	t = − .10^a^ *p* = .92	39.4 (7.5)	t = .97^a^ *p* = .33
Female	241	90.3	52.9 (24.9)		30.2 (3.9)		37.8 (8.1)	
Educational Background								
Primary education	120	45	55.4 (22.6)	F = 3.31^b^ *P* = .21	30.2 (3.9)	F = .647^b^ *P* = .59	38.0 (8.1)	F = .28^b^ *P* = .84
Lower secondary education	110	41.2	58.0 (22.9)		30.4 (3.6)		38.0 (8.3)	
Upper secondary education	29	10.9	45.3 (29.4)		30.3 (4.5)		38.1 97.4)	
Diploma	8	3	49.0 (16.3)		28.4 (3.9)		35.4 (7.1)	
Occupation								
Unemployed	102	38.2	50.6 (24.4)	F = 1.34^b^ *P* = .26	30.0 (3.7)	F = .85^b^ *P* = .47	37.8 (8.2)	F = .25^b^ *P* = .86
Student	4	1.5	37.5 (30.8)		27.8 (0.5)		37.8 (10.1)	
Employee	97	36.3	53.2 (26.5)		30.3 (4.0)		38.7 (7.4)	
Self-employed	64	24	56.8 (22.3)		30.6 (4.0)		38.7 (7.4)	
Relationship with older adults								
Children	61	22.8	50.5 (25.9)	t = − .81^a^ *P* = .42	30.0 (3.7)	t = − .47^a^ *P* = .63	37.0 (7.8)	t = -1.05^a^ *P* = .30
Other relatives	206	77.2	53.5 (24.5)		30.3 (3.9)		38.2 (8.1)	
Sharing responsibility with other in caring for older adults								
Yes	226	84.6	53.1 (25.0)	t = − .45^a^ *P* = .66	30.2 (3.8)	t = .25^a^ *P* = .80	38.2 (8.1)	t = .21^a^ *P* = .84
No	41	15.4	51.2 (24.3)		30.4 (4.3)		37.9 (8.0)	
Living in the same house with older adults								
Yes	250	93.6	52.8 (25.1)	t = − .11^a^ *P* = .92	30.2 (3.8)	t = − .53^a^ *P* = .60	38.3 (7.90	**t = 2.5** ^**a**^ ***P*** = .01*
No	17	6.4	53.4 (20.4)		30.7 (4.4)		33.2 (8.8)	
Duration of caring for older adults								
≤ 1 year	18	6.7	46.8 (28.0)	F = .79^b^ *P* = .50	30.5 (3.7)	F = .48^b^ *P* = .70	37.8 (7.4)	F = .76^b^ *P* = .52
> 1 - ≤ 3 years	115	43.1	51.4 (24.4)		30.1 (3.9)		37.8 (8.2)	
> 3 - ≤ 5 years	73	27.3	54.7 (22.9)		30.0 (3.9)		39.0 (8.2)	
> 5 years	61	22.8	55.0 (27.0)		30.7 (3.8)		37.0 (7.8)	
Duration of supporting older adult’s ADL in a day								
Undefined	116	43.4	53.1 (26.2)	F = .66^b^ *P* = .58	30.4 (3.9)	F = 1.26^b^ *P* = .29	37.4 (8.4)	F = .38^b^ *P* = .77
≤ 3 h	18	6.7	58.8 (21.7)		30.8 (4.6)		38.9 (8.2)	
> 3 - ≤ 8 h	67	25.1	50.0 (24.9)		29.5 (3.60		38.1 (7.7)	
> 8 h	66	24.7	53.5 (23.3)		30.6 (4.0)		38.5 (7.7)	
Living with older adult (s) who experienced PI(s)								
Yes	30	11.2	60.8 (18.6)	t = -1.89^a^ *P* = .06	30.8 (4.3)	t = − .91^a^ *P* = .36*	39.4 (8.7)	t = -1.06^a^ *P* = .29
No	237	88.8	51.8 (25.4)		30.2 (3.8)		37.8 (8.0)	
Had experience in PI care, n (%)								
Yes	4	1.5	56.3 (12.5)	t =-.28^a^ *P* = .78	30.2 (3.9)	t =-1.84^a^ *P* = .07	36.8 (9.0)	t = .30^a^ *P* = .77
No	263	98.5	52.8 (25.0)		33.8 (3.3)		37.9 (8.0)	

ADL = Activities of Daily Living ^a^ Independent sample t-test ^b^ ANOVA test * significant difference

### Knowledge of participants

The total score of participants on knowledge domain and scores based on their characteristics are shown on Table 1. There were no statistical differences in knowledge scores between participants based on their characteristics. Overall, the participants’ mean score was 52.8%, meaning that they could only answer half of the questions correctly. After dividing into groups of their knowledge level, looking at the answers to all knowledge questions given as shown in Table 2, more than half of participants (61%) had insufficient knowledge about PI prevention. The item ‘Pressure injury prevention in older adults related to prevent prolonged pressure on the skin’ had the lowest percentage of correct answers (24.0%), followed by the item ‘Symptoms of PIs’ (33.3%) and ‘Definition of PIs’ (37.1%), respectively.

**Table 2:** Results on the knowledge domain in per item (*n* = 267)

Item		Percentage of correct answer (%)
**Topic: Definition of PI and older adult’s characteristics**		
1	The normal changes that occur in the older adult’s skin	73.8
2	Definition of a pressure injury	37.1
**Topic: Symptoms**,** cause**,** and consequences of PI**		
3	Symptom(s) of pressure injuries	33.3
4	The cause of a pressure injury	68.2
5	Consequences of pressure injuries in older adults	47.2
**Topic: Preventive strategies that family caregivers can perform to prevent PIs**		
6	Pressure injury prevention in older adults (Prevent prolonged pressure on the skin)	24.0
7	Pressure injury prevention in older adults (Adequate feeding and drinking)	62.2
8	Pressure injury prevention for immobile/ bedridden older adults (regularly change position)	43.8
9	Pressure injury prevention in older adults (Moisturizes dry skin)	64.0
10	Pressure injury prevention in older adults (Release pressure and shear)	46.4
11	Pressure injury prevention in older adults who suffer from deep pressure injury (access health care services)	52.8
12	Pressure injury prevention in older adults (Using a special mattress)	78.3
**Category based on the knowledge score** Percentage of participants		
	Sufficient ( Total score ≥ 65%)	39%
	Insufficient ( Total score < 65%)	61%

### Attitude of participants toward PI prevention

Table 1 shows the mean score on the attitude domain of 30.2, demonstrating a positive attitude toward PI prevention. No differences in attitude scores were found between participants based on the participant’s characteristics. Almost all participants (94.4%) had a positive attitude toward PI prevention. Analyzing the results on the individual items of the attitude domain demonstrate nearly all participants (99.6%) agreed with the statement “I am responsible for the health of the older relative in my house”. Most participants (98.1%) agreed that pressure injuries in their older relatives should be prevented (See Table 3).

**Table 3:** Results on the attitude domain per statements (*n* = 267)

Statements	Strongly disagree *N* (%)	Disagree *N* (%)	Agree *N* (%)	Strongly agree *N* (%)
I am responsible for the health of the older relative in my house	0 (0.0)	1 (0.4)	143 (53.6)	123 (46.1)
The personal hygiene of the older relative in my house must be cared for carefully	0 (0.0)	4 (1.5)	144 (53.9)	119 (44.6)
I have to pay attention to the skin moisture and hygiene of the older relative in my house.	1 (0.4)	3 (1.1)	146 (54.7)	117 (43.8)
It is important to pay attention to the food and drinks of the older relative in my house	0 (0.0)	1 (0.4)	159 (59.6)	107 (40.1)
Pressure injuries on the older relative in my house should be prevented	1 (0.4)	4 (1.5)	175 (65.5)	(87 (32.6)
Helping the older relative in my house in their activities and movements is my responsibility	2 (0.7)	5 (1.9)	163 (61.0)	97 (36.3)
Immobile older relative in my house need to be helped in movement and positioning	1 (0.4)	17 (6.4)	165 (61.8)	84 (31.5)
The older relative in my house who experiences pressure injuries need to be checked by formal health care service	2 (0.7)	18 (6.7)	161 (60.3)	86 (32.2)
The older relative in my house who is at risk of getting pressure injuries needs a special mattress to prevent pressure injuries	0 (0.0)	5 (1.9)	149 (55.8)	113 (42.3)
**Category based on the attitude score** Percentage of Participants				
Positive atitude toward PI prevention				94.4%
Negative atitude toward PI prevention				5.6%

Practice of participants on PI prevention

### Practice of participants on PI prevention

Table 4 shows almost half (41.2%) of participants had inadequate practice on PI prevention. Some participants (12.7%) never helped their older relatives who were bedridden to change their position (repositioning) regularly, and some of them (24.0%) only repositioned their bedridden older relatives ‘sometimes’. The activity ‘Provide a special mattress for a bedridden older relative in my house’ had the lowest mean score (2.8), meaning that this activity was performed the least. Participants who lived in the same house with older adults had a higher practice score compared to family caregivers who were not living in the same house with older adults (38.3 vs. 33.2; t = 2.5; *p* = .01). No difference in score was found between family caregivers who took care of their relative for a longer period versus a shorter period (*p* > .05). Also, no other differences in practice scores were found between subgroups based on participants’ characteristics.

**Table 4:** Results on the practice domain per statements (*n* = 267)

Domain and item generation	Never *n* (%)	Sometimes *n* (%)	Often *n* (%)	Always *n* (%)	Mean (SD)
**Topic: Activities performed to support older adults to meet nutritional and fluid needs**,** maintain environmental hygiene**,** and access health care services**					
Provide healthy food for the older relative in my house	3 (1.1)	34 (12.7)	49 (18.4)	181 (67.8)	3.53 (0.76)
Provide mineral water for the older relative in my house at least 8 glasses in a day	16 (6.0)	61 (22.8)	60 (22.5)	130 (48.7)	3.14 (0.97)
Maintain the environmental hygiene for the older relative in my house	39 (14.6)	33 (12.4)	60 (22.5)	135 (50.6)	3.09 (1.1)
Took the older relative in my house to health services if they suffer from wounds	15 (5.6)	36 (13.5)	65 (24.3)	151 (56.6)	3.32 (0.91)
**Topic: Activities performed to support older adults in** **Mobilization**,** repositioning and facilitating a special mattress**					
Help the older relative in my house to do activities if they cannot do it him/herself	16 (6.0)	64 (24.0)	61 (22.8)	126 (47.2)	3.11 (0.97)
Help for the older relative in my house to move if they cannot do it him/herself	32 (12.0)	58 (21.7)	48 (18.0)	129 (48.3)	3.03 (1.09)
Help the bedridden older relative in my house to change their position (positioning) regularly if they cannot do it him/herself	34 (12.7)	64 (24.0)	56 (21.0)	113 (42.3)	2.93 (1.11)
Provide a special mattress for a bedridden older relative in my house	45 (16.7)	57 (21.3)	61 (22.8)	104 (39.0)	2.84 (1.12)
**Topic: Activities performed to support older adults in skin** **hygiene and moisture care**					
Prevent the older relative in my house from using damp and wet clothes, including changing diapers regularly (if they use diapers)	26 (9.7)	44 (16.5)	66 (24.7)	131 (49.1)	3.13 (1.02)
Prevent long pressure on the body of the older relative in my house	42 (15.7)	49 (18.4)	62 (23.2)	114 (42.7)	2.93 (1.11)
Moisturize the skin of the older relative in my house by giving lotions/oils	3 (1.1)	18 (6.7)	71 (26.6)	175 (65.5)	3.57 (0.67)
Check the entire skin of the older relative in my house for redness	7 (2.6)	38 (14.2)	83 (31.1)	139 (52.1)	3.33 (0.82)
**Category based on the total score** Percentage of Participants					
Adequate practice on PI prevention					58.8%
Inadequate practice on PI prevention					41.2%

1) ‘never’ if the activity has never been done; (2) ‘sometimes’ if the activity is done 1 to 3 days per week; (3) ‘often’ if the activity is done 4 to 6 days per week; and (4) ‘always’ if the activity is done every day

### Correlation between knowledge, attitude and practice

The correlation analysis between domains is shown in Table 5. The knowledge and attitude toward PI prevention appeared to be positively correlated to practice on PI prevention, with the correlation value of X^2^ = 003; X^2^ = 9.887 and the significance value of *p* = .45 and *p* = .002, respectively. Individual analysis of participants showed lower practice scores in the participants who also had a lower knowledge score. Family caregivers who knew about strategies to prevent PI, in specific, gathered a higher total practice score. No correlation was found between knowledge and attitude (*p* > .05).

**Table 5:** Correlations for study variables (Crosstab X^2^)

Variable	1	2	3
1. Knowledge			
2. Attitude	0.007		
3. Practice	4.003*	9.877*	

* *p* < .05 (significant correlation)

## Discussion

The objectives of this study were to measure knowledge, attitude and practice of family caregivers on PI prevention among their older relatives, in the capital city of West Java, Indonesia by using the newly developed and valid instrument named “Knowledge, Attitude and Practice of Family Caregivers at Preventing Pressure Injuries (KAP-PI)”. The results showed that 61% of family caregivers had clear deficiencies in knowledge (less than 35% of all questions were answered correctly), almost all participants (94.4%) had a positive attitude toward PI prevention and nearly half (41.2%) of participants had inadequate practice on PI prevention. Knowledge and attitude toward PI prevention correlated positively with practice on PI prevention, but no correlation was found between knowledge and attitude.

Family caregivers can be a strategic support system for preventing PI problems at home, especially for countries where taking care of parents to the end of their lives is conventional and mostly is done by the family. The present study explained that the majority of family caregivers cared for their older relatives for more than 3 years and more than 3 h per day. It was expected that the length of experience in caring for the older adults would make the family caregivers aware of their health problems [36]. However, the current study found that there were no differences on the knowledge, attitude and practice scores measured between those who have taken care of their older relative for longer period versus shorter period. A reason could be that research had shown that none of the participants had received any information about PIs or PI care from formal health care providers even though they received or made contact with formal care [10, 37].

Family caregivers lacked knowledge about PI prevention. Similar results were found by Tavares et al. (2016), who also found a knowledge deficit about PI care among family caregivers of older adults admitted to the emergency unit in Brazil [12]. An early study, performed by Baharestani (1993), studying the life experiences of women in the United States caring for their frail homebound older husbands with PIs, already showed limited knowledge on PI prevention and wound treatment [38]. Last two studies among caregivers to the patients admitted to a tertiary hospital in South India [39] and Sohag Governorate Hospitals Egypt [40] revealed the same issue that family caregivers lack of knowledge about PI prevention. In the current study, only approximately 30% of family caregivers knew the definition and symptoms of PIs. Information regarding ‘preventing prolonged pressure on the skin can prevent PIs’ was only known by a small number of family caregivers (24%). Following this, some participants (12.7%) in this study stated that they never helped their older relatives who were bedridden to change their position (repositioning) regularly. Furthermore, most participants did not consider repositioning and releasing pressure to successfully prevent pressure injuries. Consequently, 24% of participants performed the repositioning activity for their bedridden older relatives only ‘sometimes’. While, repositioning is strongly recommended for those at risk of PI, such as bedridden older adults [1, 41]. As repositioning should be arranged on an individualized schedule for older adults with or at risk of Pis [1], it is crucial for family caregivers to be near older adults. The current study found that family caregivers who live in the same house as the older adults had higher practice score on PI prevention.

When looking at the attitude of family caregivers towards PI prevention, family caregivers generally demonstrated a positive attitude. As an example, they strongly agreed to the statement “I have to pay attention to the skin moisture and hygiene of the older relative in my house”. Likewise, they mostly agreed that “Helping the older relative in my house in their activities and movements is my responsibility”, and “The immobile older relative in my house needs to be helped in movement and positioning”. These results show a high sense of familial responsibilities among family caregivers in Indonesia to support their older relatives. Similar positive attitudes regarding taking care of older relatives, in general, were also discovered among later-generation Chinese-American caregivers in Seattle and Houston [42]. Their responsibilities towards parents do not change over time, although they experience acculturation. Our study strengthens the fact that people in Asian countries are commonly accountable for taking care of their parents. To maintain such a positive attitude, adequate support from the healthcare system, such as increasing knowledge, is essential [26, 43]. Providing family caregivers with the knowledge needed to translate the positive attitude into effective prevention behaviors in practice is fundamental [43], which is insoluble without adequate support and coaching from professional caregivers.

This study found significant statistical correlations between knowledge and attitude domains with practice domains. Individual analysis of participants showed lower practice scores in the participants who also had a lower knowledge score. The higher the knowledge score was, the stronger the practice on PI prevention. This finding added to evidence that good practice should be built through good knowledge [21]. Furthermore, this study agrees that it is necessary to investigate attitude because attitude is a predictor of practice, and attitude does not always correlate with knowledge [24, 43]. However, a study to get more insight into family caregivers’ barriers in performing PI prevention and how they can be supported by professional care providers would be helpful. Research should also be conducted to explore the necessary professional support to strengthen family caregivers’ role in PI prevention. Taken into a priority, the knowledge, attitude and practice assessment in this study indicated the need for PI prevention education for family caregivers of community-dwelling older adults in Indonesia.

### Implication for practice and research

This study is the first in Indonesia to successfully address family caregivers’ knowledge, attitude, and practice on PI prevention. A previous research showed that prevalence of PI in this population calls serious attentions [10]. As the number of older adults living at home in Indonesia is high and considering characteristics of family caregivers in Indonesia, a strategy to address this problem should become a priority. Building a collaboration between formal and informal care to address the PI problem in Indonesian community-dwelling older adults might be a good solution. Family caregivers in this study strongly tended to take care for their older relatives which could be a favorable aspect for formal caregivers to plan an intervention involving family caregivers. In addition, further research should focus on which education program and educational materials and practice support are appropriate for them to solve PI problem among Indonesian community-dwelling older adults.

### Study limitations

This study was the first cross-sectional study about PI prevention involving family caregivers in the general population. The study was conducted in Bandung city, the capital city of the largest populated province in Indonesia. However, family caregivers’ knowledge, attitude, and practice in pressure injury prevention may vary regarding different regional characteristics. Hence, generalizing the findings should be done with caution. Furthermore, direct observation might be more accurate to assess the family caregiver’s practice, which was not done in this study.

## Conclusion

This study shows that family caregivers in a large city in Indonesia had considerable knowledge deficiencies and inadequate practice on PI prevention. Knowledge and attitude positively correlated with practice. The higher the knowledge score was, the stronger the practice on PI prevention. Positive attitude toward PI prevention among family caregivers strengthen the fact that Indonesian people tend to care for their older relatives. Health care professionals should prioritize educating and coaching family members on preventive strategies for PIs, while further research should identify effective education programs, materials, and support practices to address PI issues among Indonesian community-dwelling older adults.

## Data Availability

All data generated or analysed during this study are included in this published article. The raw data is available from the corresponding author on reasonable request.

## Abbreviations

PI: Pressure Injury
PIS: Pressure Injuries
KAP-PI: Knowledge, Attitude, and Practice on Pressure Injury
IBM SPSS: International Business Machines Statistical Package for the Social Sciences
ADL: Activities of Daily Living
